# Accuracy of additive manufacturing in stomatology

**DOI:** 10.3389/fbioe.2022.964651

**Published:** 2022-08-16

**Authors:** Yao Tang, Yunfan Zhang, Zhaoqiang Meng, Qiannan Sun, Liying Peng, Lingyun Zhang, Wenhsuan Lu, Wei Liang, Gui Chen, Yan Wei

**Affiliations:** ^1^ Department of Orthodontics, Cranial Facial Growth and Development Center, Peking University School and Hospital of Stomatology, Beijing, China; ^2^ NMPA Key Laboratory for Dental Materials, National Center of Stomatology, National Clinical Research Center for Oral Diseases, National Engineering Research Center of Oral Biomaterials and Digital Medical Devices, Research Center of Engineering and Technology for Computerized Dentistry Ministry of Health, Beijing, China; ^3^ Department of Geriatric Dentistry, Peking University School and Hospital of Stomatology, Beijing, China

**Keywords:** three-dimensional (3D) printing, digital manufacturing, dentistry, maxillofacial surgery, precision, trueness

## Abstract

With the rapid development of the three-dimensional (3D) printing technology in recent decades, precise and personalized manufacturing has been achieved gradually, bringing benefit to biomedical application, especially stomatology clinical practice. So far, 3D printing has been widely applied to prosthodontics, orthodontics, and maxillofacial surgery procedures, realizing accurate, efficient operation processes and promising treatment outcomes. Although the printing accuracy has improved, further exploration is still needed. Herein, we summarized the various additive manufacturing techniques and their applications in dentistry while highlighting the importance of accuracy (precision and trueness).

## 1 Introduction

Three-dimensional (3D) printing, also known as additive manufacturing (AM) (Gebhardt et al., 2019a), is a new technology that has been developed over the past few decades. The technology is based on the principle of layered manufacturing, in which printers read data from computer-aided design (CAD) and materials (liquid, powder, or flake) overlap layer by layer to form dense three-dimensional objects. In 1981, Hideo Kodama described the process of 3D printing for the first time in the manufacture of 3D plastic models at the Nagoya Industry Research Institute (Kodama, 1981). Since then, 3D printing process has been maturing gradually. Diverse types of materials involving polymers, ceramics, metal, and composite materials have been applied in 3D printing nowadays (Ladd et al., 2013; Sun et al., 2013; Scheithauer et al., 2015; Ligon et al., 2017), which brings benefits into areas such as space engineering, construction industry, and various medical applications including surgery and dentistry (Tay et al., 2017; Anupama and Rachel, 2018; Kelly et al., 2018; Contessi Negrini et al., 2020).

Compared with the traditional manufacturing process, 3D printing has attracted broad attention in the biomedical field for two main reasons: first, the inherent advantages of the technology, such as versatility, ease of use, accurate control of the manufacturing process, and higher material utilization ratio, and then, the customized product is highly favored for its characteristics of shape and structure conforming to a biomedical application prospect (Murphy and Atala, 2014; Kim et al., 2016). In recent years, the application of 3D printing in the medical field has gradually expanded. The 3D printing industry has made huge strides in medical devices, implantable materials, and cell printing due to lower manufacturing costs but improved printing accuracy and speed. 3D printing has also been applied in pathological organ models to assist preoperative planning and surgical treatment analysis, personalized manufacturing of permanent implants, preparation of fabricating local bioactive and biodegradable scaffolds, and even directly printing of tissues and organs with complete life functions (Gross et al., 2014; Yan et al., 2018; Nguyen et al., 2019).

Different from other tissues and organs in the human body, teeth and craniofacial bones also possess unique esthetic properties. Since they are small and highly irregular, clinical operations and devices should always obtain a precise match on a microscale in order to achieve outstanding treatment results. Moreover, rich networks of vital blood vessels and nerves gather in the maxillofacial region, which makes medical procedures extremely risky. Thus, as a subject that involves specifically complex structures of oral soft and hard tissues and requires particularly high precision and accuracy, stomatology can gain great convenience from 3D printing on personalized and precise diagnosis and treatment. Through 3D printing technology, based on imaging data such as magnetic resonance imaging (MRI) or computed tomography (CT) of patients, rapid manufacturing of personalized scaffolds, preparation of organ models, or direct printing of defects can be completed (Gross et al., 2014; Murphy and Atala, 2014; Nguyen et al., 2019). Therefore, this review intends to connect the various 3D printing technologies with their applications in dentistry and discuss the importance of accuracy in 3D printing, the current challenges, and further perspectives as well.

## 2 3D printing processes

**FIGURE 1:**
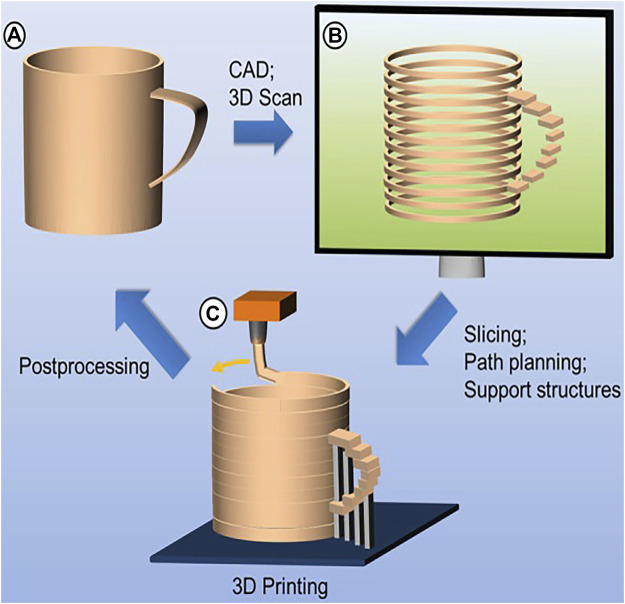
Basic principles of additive manufacturing. **(A)** Data acquisition; **(B)** data processing; **(C)** printing (source: Ligon et al. 2017)

3D printing is mainly divided into three steps: data acquisition, data processing, and printing (as shown in Figure 1). At present, many kinds of 3D modeling and scan imaging methods are used in the process of 3D printing, including magnetic resonance imaging (MRI), computed tomography (CT), the 3D scanner, CAD, and computer-aided engineering (CAE) (Bilgin et al., 2015; Cantín et al., 2015; Jung et al., 2016; Yahata et al., 2017). The obtained data are imported into a reconstruction software application to guide the printer to print the target part. Based on different working principles, many 3D printing approaches have emerged, including the stereolithography apparatus (SLA), fused deposition modeling (FDM), selective laser sintering (SLS), selective laser melting (SLM), laminated object manufacturing (LOM), and digital laser processing (DLP) (Ligon et al., 2017; Gebhardt et al., 2019b; Hernandez et al., 2019; Shakor et al., 2019; Zhou et al., 2020) (Table 1)

**TABLE 1:** Available 3D printing processes.

3D Process	Material	Mechanism	Need supports in the process?	Layer thickness	Advantages (Lin et al., 2019)	Disadvantages
SLA	Photosensitive	Polymerization	Yes	0.02–0.2 mm Melchels et al. (2010); Zakeri et al. (2020)	Fast and high accuracy	Limited material
PolyJet	Photosensitive	Polymerization	Yes	0.016 mm Kasparova et al. (2013); Lee et al. (2015); Park and Shin (2018); Liu et al. (2020)	Smooth surface and high accuracy	High cost and short service life
SLS/SLM	Powders: wax, plastic, metal, and ceramic	Sintering/melting	Yes/no	0.05–0.15 mm (Torres et al. (2011); Tan et al. (2017); Awad et al. (2020)	Good mechanical properties and increasing machinable materials	Low accuracy, low efficiency, limited quality, and high cost
FDM	Thermoplastic	Extrusion	Yes	0.1–0.3 mm Turner and Gold (2015); Ozbolat and Hospodiuk (2016); Blok et al. (2018)	Efficient, environmental-friendly, and cheap	Low accuracy, tough surface, and high-temperature process

### 2.1 SLA and DLP

Laser stereoscopic lithography, also known as the stereoscopic apparatus, is the most widely researched, mature, and until today the widely used 3D printing technology. With the help of the ultraviolet laser beam, liquid monomers are converted to solid state through polymerization, which can produce parts with excellent surface quality and fine detail (Kim et al., 2017a). Under the control of computer, the lithography machine coats the resin on the surface of the part (*x-y* direction), and the laser scanner controls the laser beam to move and scan point by point within the cross-sectional profile according to the layered contour data of the CAD model; once the beam penetrates the surface of the resin, instantaneous solidification by polymerization is realized, and then, the platform will be lowered by the amount of the specific layer thickness to provide curing of each layer and connection to the previous layer.

SLA shows many advantages such as the highly automated printing process, high-dimensional accuracy with the smallest details of 0.02–0.2 mm (Melchels et al., 2010; Zakeri et al., 2020), extraordinary speed, excellent surface quality, and high resolution to produce complex parts. But the parts are easy to bend and deform due to materials’ limitation; therefore, the building process requires support which limits the options for the orientation of the parts as the support will leave marks on the surface of the parts after removal. Then, a smaller range of materials is available, especially resins, which now also contains nanoparticles made of carbon or ceramic materials. This technique is most commonly used to print dental or bone models for diagnosis and treatment design and is also applied to manufacture clear aligners, occlusal veneers, denture teeth, and customized surgical guides (Mavili et al., 2007; Jindal et al., 2019; Cha et al., 2020; Ioannidis et al., 2020).

**FIGURE 2:**
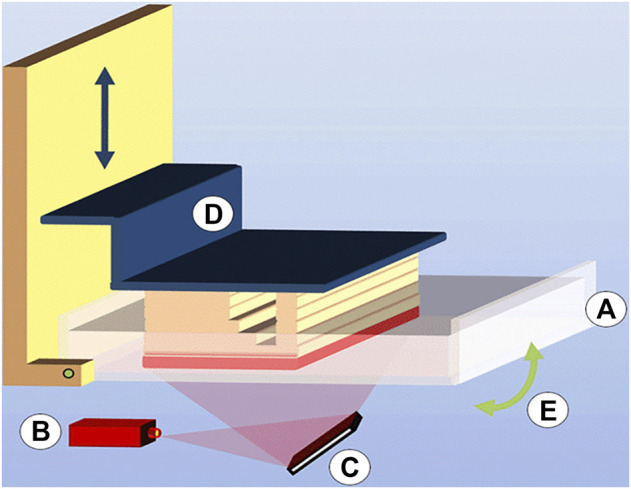
Digital light processing (DLP) consisting of **(A)** a vat filled with photopolymer resin, **(B)** light source, **(C)** micromirror array, **(D)** vertically movable building platform, and **(E)** tilting device to replenish the uncured bottom layer (source: Ligon et al. 2017).

DLP technology is similar to SLA technology, and it also uses the characteristics of photosensitive material to polymerize and solidify under ultraviolet light irradiation (Quan et al., 2020) (as shown in Figure 2). But its speed is much faster than SLA as DLP technology uses a digital light projector to cast ultraviolet light, which can directly allow any selected portion of the entire x–y workspace to be exposed simultaneously and speed cycle times between layers (Stansbury and Idacavage, 2016; Revilla-León et al., 2018).

### 2.2 SLS and SLM

**FIGURE 3:**
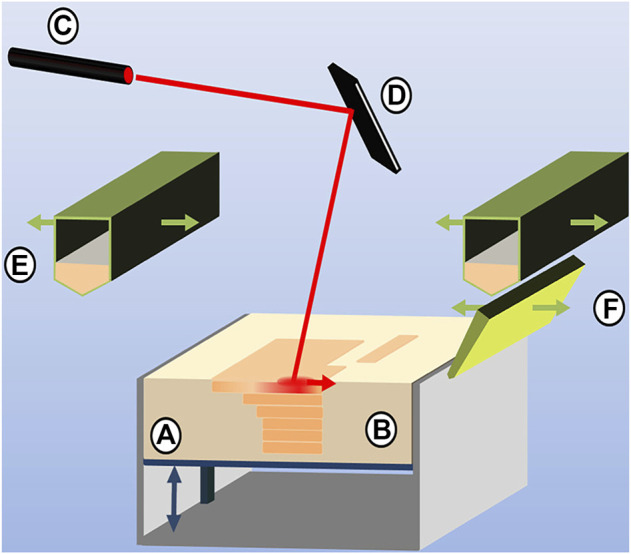
Selective laser sintering process composed of **(A)** vertically movable build platform, **(B)** powder bed with embedded, sintered model layers, **(C)** laser source and **(D)** laser optics, **(E)** powder feedstock and deposition hopper, and **(F)** blade for powder distribution and leveling (source: Ligon et al. 2017).

It is a manufacturing technology often used to process powder materials (Hussain et al., 2019). This technology uses the laser beam as a heat source under the control of a galvanometer and is guided by computer-aided design data to melt the selective area of the powder layer by layer. As the beam continues to move, the melted part solidifies to produce a solid layer due to the heat transfer by thermal conductivity to the surrounding powders, and then, the next layer is sintered, and the layer-to-layer bonding is realized (as shown in Figure 3). On account of making good use of metal material, SLS/SLM has advantages in manufacturing customized brackets, removable partial dentures, and dental implants (Wiechmann, 1999; Jiang et al., 2019; Yang et al., 2019).

Compared with the conventional working process, the process of SLS has strongly shown the advantages of simple operation and produces molds with high hardness. Without support, the unsintered powder plays a supporting role during the process. But there are still some shortcomings: 1) the material needs to be preheated and cooled, and postprocessing is troublesome; 2) the sintered material has many voids that lead to poor density and mechanical properties; 3) the surface of the part is rough and porous with the layer thickness of 0.05–0.15 mm, which can be improved by postprocessing (Murr et al., 2009; Torres et al., 2011; Tan et al., 2017; Awad et al., 2020); and 4) it costs a fortune to fill the processing room with nitrogen to ensure sintering safety.

SLM is developed on the basis of SLS for manufacturing metal parts with very accurate density, and the mechanical properties of SLM products are comparable to those of the conventional products (Gebhardt et al., 2019b). Laser melting is very similar to the abovementioned laser sintering process, except that during the printing process, the powder material is completely melted by the laser to produce a local (selective) melt pool, which results in complete dense parts with good mechanical properties after solidification. However, the surrounding powders cannot provide sufficient support due to the weight of the material and the limitations to the printing process; thus support is required during the SLM process.

### 2.3 FDM

This process is suitable for thermoplastic materials such as acrylonitrile butadiene styrene (ABS) and polylactic acid (PLA) (Rocha et al., 2014), which can produce color parts by using color materials with dimensional accuracies typically in the order of 0.1 mm (Turner and Gold, 2015; Ozbolat and Hospodiuk, 2016). From a technical perspective, FDM is an extrusion process that is formed by single-layer contour superposition. Among the 3D printing technologies, the design of FDM is the simplest and is currently widely used. An FDM machine can work without a laser and consists of a sealed and heated construction space (approximately 80°C for ABS), equipped with an extrusion head and a build platform. After being fed into the extrusion head, the filamentous thermoplastic material is partially melted by electric heating and then extruded through the nozzle. Under the guidance of the contour information, the nozzle moves in the *x*-*y* direction, while the build platform moves in the *z* direction. As the molten material cools, the bonding between layers is realized until the parts are finished. The build process requires the addition of supports, which are generated through a second nozzle and can be added simultaneously with the molded material using different plastic materials. However, limited control over the placement of the material and the creation of voids adversely affect its accuracy, especially when printing more complex shapes (Blok et al., 2018).

The main advantages of FDM are as follows (Lin et al., 2019): 1) simple principle and operation, and low cost; 2) use of color materials; 3) unique soluble support technology which can achieve complex geometric shapes and internal cavities; 4) nontoxic and environmental-friendly materials; 5) faster forming speed; and 6) simple postprocessing. Shortcomings include the following: 1) low accuracy; 2) stripe formation on the forming surface of the parts due to limitations to the printing process; 3) longer printing time because of the need for design and production of supports; and 4) requirement of high-temperature control in the printing process; if the temperature is too low, the material will not melt completely, while the material will not be easily formed with excessive temperature. Thus, it allows for the printing of crude anatomical models without too much complexity.

### 2.4 PolyJet

**FIGURE 4:**
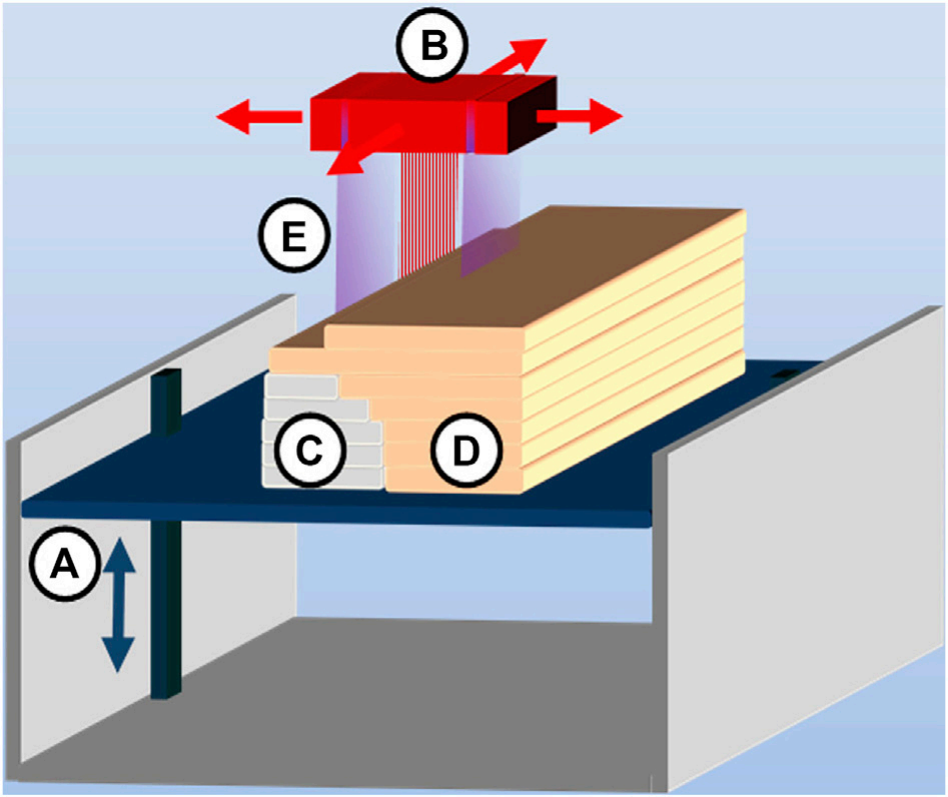
PolyJet process consisting of **(A)** vertically movable building platform, **(B)** multinozzle inkjet head, **(C)** layers of support material, **(D)** layers of building material, and **(E)** UV source attached to an inkjet head (source: Ligon et al. 2017).

Generating parts by ultraviolet solidifying of liquid monomers, PolyJet represents the SLA process in principle more (Cohen et al., 2009). The design of the machine is similar to that of a 2D office printer (as shown in Figure 4), the material is directly applied to the build platform by means of a multiple-nozzle piezoelectric printing head, and the ultraviolet light lamp moves to solidify the material. The layer's middle thickness is only 0.016 mm (Liu et al., 2020), and the product details are precise, providing a very smooth surface. Thus, PolyJet can manufacture more precise models and customized guides than SLA (Hazeveld et al., 2014). PolyJet can use materials with a variety of colors and different shore hardness ranges because of its multiple print heads so as to realize full-color digital printing (Hofmann, 2014). At the same time, due to the use of multiple sprinklers, parts can be printed with water-soluble or hotmelt materials to add supports, which can be washed during the automatic finishing process without leaving traces on the parts (Murugesan et al., 2012).

Compared with other 3D printing technologies, PolyJet exhibits several advantages (Salmi et al., 2013): 1) it can print different materials at the same time, which can meet different colors, transparency, and stiffness requirements; and 2) the processing precision is high, with the printing layer thickness as low as 16 microns. In contrast, the disadvantages are as follows: 1) the product is usually suitable for short service life; 2) the processing precision is expensive; 3) the strength of the finished product is low, and 4) a specially developed photosensitive resin is required to increase the strength.

## 3 Application of 3D printing in the oral clinic

### 3.1 3D-printed dental model

Compared with the traditional plaster model, 3D-printed model has higher precision and trueness and is not easy to wear out. Meanwhile, it is more convenient for storage and viewing. The application of scanning technology reduces time and discomfort caused to patients when taking impressions (Yuzbasioglu et al., 2014). Nowadays, it is increasingly common to simulate and analyze craniofacial structures by printing 3D models in various oral clinics, and computer-assisted surgery is now regularly used to improve the prediction of the results of maxillofacial surgery. Mavili et al. (2007) have proved that using life-size 3D-printed models can improve the accuracy of orthognathic surgery and prevent condylar process dislocation and sagging.

In addition, orthodontic clinicians need to make judgments and further formulate and modify treatment plans based on the arrangement of teeth and bite conditions at different stages of treatment. With the application of digital technology, it is possible to convert gypsum models or impressions into 3D digital models directly, but the digital models cannot completely replace the role of the physical model in the subsequent fabrication of the appliance and analysis of the treatment effect. Park and Shin (2018) and Kasparova et al. (2013) conducted comparative studies between 3D-printed dental models and traditional plaster dental models and found that the comprehensive performance of 3D-printed dental models was better than that of traditional plaster dental models, and 3D-printed models can replace the traditional plaster models for orthodontic clinics. Some scholars even thought that plaster models might be redundant for orthodontic treatment because 3D digital models could be used at all stages of the whole orthodontic workflow (van der Meer et al., 20161939).

But to use 3D-printed dental models for clinical purposes, accuracy of the printed outcome must be prescribed tolerances. Errors can occur and accumulate at each step in the process: scanning, segmentation, CAD modeling, 3D printing, and postprocessing (Szymor et al., 2016). Even when the same model is printed, the accuracy of the resulting part depends on the 3D-printer technology and machine used. Previous research studies (Hazeveld et al., 2014; Brown et al., 2018; Kim et al., 2018) have measured dental models made by different 3D printing technologies and came to the conclusion that the DLP and PolyJet were more precise than the SLA and FDM, and all of them can meet clinical requirements. The thickness difference (FDM: 0.330 mm, DLP: 0.050 mm, and PolyJet: 0.016 mm) of the layers may have affected the results. The study by Lee et al. (2015) shows similar results; mean deviation measurements with best-fit alignment of FDM and PolyJet groups were 0.047 and 0.038 mm, and the dimensions of controlled STL files were closer to the PolyJet replicas than to the FDM replicas. However, 3D printers should be selected based on the trueness and precision required for the specific orthodontic appliance; invisalign aligners (Align Technology, Santa Clara, CA) consist of several aligners with maximum tooth movement in each aligner from 0.25 to 0.3 mm (Wang et al., 2019). Thus, the accuracy of dental models must be smaller than 0.25–0.3 mm for the fabricated aligner to exert an orthodontic force on the teeth; PolyJet may have more advantages in terms of accuracy.

### 3.2 Customized brackets

Recently, customized orthodontic appliances including metal labial or lingual brackets and clear aligners have been developing rapidly and receiving growing attention, which has caused a tremendous threat to traditional brackets (Fujiyama et al., 2014). Yang et al. (2019) used a high-resolution rapid digital light processing (DLP) technology printer to convert the virtual bracket models into wax patterns and made polycrystalline alumina ceramic brackets based on them, making it possible to realize mass customization for ceramic orthodontic brackets. But additive manufacturing still needs more improvement in terms of dimensional accuracy, surface quality, and the mechanical properties in order to allow fabricating customized ceramic brackets directly.

Since proposed in the 1970s, lingual orthodontic technology has attracted more and more attention in orthodontic treatment (Fujita, 1979; McCrostie, 2006). Due to the high accuracy requirements, the emergence of 3D printing technology is no doubt a great help for the development of orthodontics. Wiechmann (1999) used the SLM technology combined with the CAD/CAM technology to print out the first personalized lingual bracket in 1999, which opened a new chapter of the lingual orthodontic. Traditional lingual brackets are rough and inaccurate, and the lack of close contact between the brackets and the teeth makes the periodontal tissue more susceptible to irritation. At the same time, patients can feel obvious foreign body sensation and are more likely to have complications such as swelling and pain in tongue and pronunciation disorder due to the large thickness of the brackets (Miyawaki et al., 1999). However, the lingual brackets produced by 3D printing technology generally adapt thinner material, which has a high degree of anastomosis with the patient’s lingual teeth face, accurate adhesion positioning, less adhesive use, simple operation, and better clinical results (Yang et al., 2019). Moreover, from the perspective of long-term clinical feasibility, the brackets are not easy to fall off. The lingual brackets are positioned on the lingual face of the teeth; it is difficult to directly bond the brackets due to the oral environment and the shape of the teeth. The treatment procedure can be greatly simplified by the precise positioning of the bracket with the transfer jigs. Fillion (2018) uses 3D printing technology to make the transfer jigs on the model and places the lingual bracket with well-positioned accuracy.

### 3.3 Aligners

At the beginning of this century, commercial invisible appliances have been successfully developed at home and abroad. An increasingly mature system is recognized by orthodontists, and the esthetic and comfortable experience it brings is also favored by adult patients (Rosvall et al., 2009; Bai, 2010). The traditional invisible appliance is based on the impression of the patient’s plaster model or 3D-printed models and is made by the hot-pressing technology (Brown et al., 2018), in which exists a large geometric error, and due to the thermoplastic nature of the material, the compressive strength of the appliance is weak, and it is easy to undergo irreversible deformation. Thus, the patient needs to remove and wear it repeatedly during different periods of eating, chewing, and activities. Just like designing and producing a retainer by the 3D printing technology (Nasef et al., 2014), it is now possible to eliminate all the intermediate steps that affect the accuracy and print the appliance directly, which makes a significant contribution to personalization, accuracy, beauty, and comfort of invisible treatment. Based on dental long-term (LT) resin, Jindal et al. (2019) and Jindal et al. (2020) printed invisible appliances, which have superior geometric accuracy, load resistance, yield, stiffness, and deformation resistance compared with traditionally thermoformed Duran appliances. In addition to biocompatibility and sufficient mechanical strength, dental LT resin shows superior accuracy and time-saving features, providing an excellent alternative to the conventional materials for manufacturing clear dental aligners.

### 3.4 Maxillofacial surgery

In the field of maxillofacial surgery, 3D printing has been widely used in many aspects such as mandibular reconstruction, facial reconstruction, skull surgery, orthognathic surgery, and temporomandibular joint reconstruction (Steinbacher, 2015; Ritschl et al., 2016; Louvrier et al., 2017; Roos et al., 2020). In addition to the anatomical model mentioned earlier, 3D objects increasingly served as surgery (cutting, drilling, and positioning) guides, occlusal splints, personalized implants (bone plates, bone reconstruction components, etc.), and facial epithelium (Louvrier et al., 2017). Ritschl et al. (2016) performed nasal-alveolar bone molding (NAM) on patients with cleft lip and palate by 3D printing NAM guides; the postoperative effect was not significantly different from conventional techniques, although it saved time and labor. 3D printing technology can be applied in the treatment of maxillary tumors; the doctor can accurately remove the diseased tissue with the customized surgical guides and repair extensive defects using customized 3D printing prostheses, which proved to be successful both functionally and cosmetically (Roos et al., 2020). Steinbacher (2015) reported a number of cases, including skull reconstruction of frontal defects realized by 3D-printed titanium mesh, 3D-printed cutting guide to assist mandible reconstruction with free fibula flap, and personalized splint to assist orthodontic surgery; all achieved satisfactory results. It can be seen that 3D printing and virtual surgery have significant contributions to improving the efficiency, accuracy, creativity, and repeatability of craniofacial surgery.

#### 3.4.1 Personalized root implants

At present, inserting dental implants is a prevailing approach to restore missing teeth. However, cone-shaped or columnar implants that do not match the shape of the tooth extraction socket are mostly used in the oral clinic, which results in the failure of primary stability of the implant after implantation into alveolar bone. Moreover, traditional pure titanium implants generally have the problems of stress-shielding and low biological activity caused by excessive elastic modulus, which greatly reduces the implantation success rate (Chrcanovic et al., 2015). Personalized root implants have been proposed in recent years; due to their excellent anti-rotation performance, instant implantation, good biocompatibility, and other advantages, they are believed to well simulate the force transfer characteristics and root stress distribution characteristics of natural teeth and indeed achieve the desired clinical effects (Chen et al., 2014; Ritter et al., 2014). With the application of 3D printing, it is possible to realize personalized immediate implants in oral clinics, using 3D-printing technology to prepare dental implants and improve the implant material composition and structure to achieve better bone induction, and the osseointegration effect has become a research hotspot in the field of stomatology. Previous studies (Guo et al., 2019; Zhao et al., 2021) have shown that the zirconia (ZrO_2_) ceramic dental implant abutment prepared by the DLP 3D printing technology can meet the requirements of dental implant materials. Metal implants with interconnected pore structures have the potential to promote the endogenous growth of bone and reduce the possibility of mismatched stiffness between the implants and the bone, thus eliminating the stress-shielding effect and achieving a better bone healing effect on the porous implant surface (compared with the solid implant surface) (Shah et al., 2016).

#### 3.4.2 Surgical guides

When transferring the virtual implant position to a model situation, guided surgery gives even experienced surgeons significantly higher predictability and accuracy than freehand surgery (Vermeulen, 2017). As early as 1987, Edge (1987) had proposed to realize accurate implants by means of the implant guide made by vacuum hot-pressing technology, which has certain limitations in the case of multiple missing teeth (Cristache and Gurbanescu, 20172017). As a static navigation method commonly used in implant surgery, a personalized implant guide can accurately transfer the preoperative design to the operation and improve the survival rate of implants (D’haese et al., 2017). Studies (Reyes et al., 2015) have shown that 3D-printed implant guides are more accurate in assisting the implant of missing teeth compared with the traditional implant guides. Van Assche et al. (2010) found that using CT data to construct a 3D-printed personalized implant guide can achieve minimally invasive surgery without flap surgery, and postoperative implant implantation accuracy can meet clinical requirements. Research by Jiang et al. (2019) has shown that 3D-printed personalized implant guides provided accurate and scientific dental implants for patients with missing anterior teeth, but due to the use of photopolymerization resin material, the model fails to simulate the elasticity of mucosal tissue; clinicians need to accurately assess the patient’s mucosa and jaw condition before surgery. Bilhan et al. (2015) also believed that the resin guide plate has great shortages such as low accuracy, insufficient harness, poor permeability, relatively expensive price, easy to deform during production process or at high temperature, and easy to deform or even break during the implant operation. Based on this, the 3D-printed composite implant guide can reduce the implant offset on the basis of ensuring the treatment effect and patient satisfaction, which indicates that it has higher application value in implant restoration, and the PolyJet system had better accuracy and reduced printing time (difference between the 3D CAD model and the printed parts was 0.03 ± 0.08 mm) (Kim et al., 2020). In Keßler et al. (2021), all surgical guides were printed with a layer thickness of 50 μm, and no significant difference was found regarding the accuracy between the surgical guides that were manufactured by 3D printing or with a milling device; the displacement of implants when using 3D-printed surgical guides appeared to be within safe ranges.

But there are some insurmountable shortcomings in the implementation of 3D-printed composite implant guides that are yet to be overcome. For example, there are some personal errors and systematic errors unavoidable in all aspects of the guide production process. In addition, the increase in the volume of the implant machine increases the difficulty of operating in the application to patients with limited mouth opening, which enlarges the accuracy error of implant positioning to a certain extent. Therefore, the guide needs to be further improved and perfected.

### 3.5 Dental restorations

The joint application of oral digital impression technology and 3D-printing technology is currently a hotspot in the field of dental prosthetics. The combination of the two has been successfully applied to zirconia all-ceramic restorations, metal restorations, wax restorations, metal racks of removable partial dentures, maxillofacial prostheses, and complete dentures (Torabi et al., 2015).

Although the anatomical structure in the mouth is complex and fine and the dentures made by traditional impression methods and restoration techniques are still insufficient in terms of tightness, it is difficult to obtain precision impressions for making dentures through traditional methods in the clinic, especially when treating patients with severely restricted mouth. Now intraoral scanning, computer-aided design, and 3D printing provide alternative methods for manufacturing dentures. Previous studies (Janeva et al., 2018) have confirmed that the stent prepared by the CAD/CAM technology can obtain a relatively ideal position and has higher precision than that of the traditional casting method. Some scholars also evaluated the mechanical properties of the 3D-printed removable stent of cobalt–chromium alloy, confirming that its elongation, tensile strength, and yield strength can meet the requirements of removable partial denture (Hassan et al., 2017). Meanwhile, the wear resistance of 3D-printed resin dentures is equivalent to that of conventional prefabricated resin dentures (Cha et al., 2020). Wu et al. (20171939) successfully designed and made removable partial dentures for patients with mouth opening difficulties by integrating intraoral scanning, computer-aided design, and 3D printing. However, in spite of advances in technology and dental materials, traditional cast partial dentures are still manufactured by waxen technology rather than 3D printing; using 3D printing technology to restore teeth, function, and esthetics is full of obstacles because knowledge, skills, and technology are needed (Fathi et al., 2016). Most of the research studies on the production of dentures by CAD/CAM and 3D printing technology have just started, and there are few cases reported by controlled studies or with a long-term follow-up, while most of the clinical cases end at the clinical trial stage.

In terms of accuracy in milling and 3D printing, studies of definitive complete dentures have reported contradictory results, some studies have found milled complete dentures were superior to the 3D-printed complete dentures (Kalberer et al., 2019; Hsu et al., 2020), some came to the opposite conclusion (Hwang et al., 2019), and some have found comparable results for both (Yoon et al., 2018). However, the conclusions of these studies are derived from the comparison of milling with only one 3D printing procedure. Herpel et al. (2021) took into account the variety of different manufacturing processes; 3D printing was less true than milling by 17–89 μm and less precise by 8–66 μm in terms of complete denture, which was within a clinically acceptable range.

In the field of fixed prosthesis, the marginal fitness of the crown is closely related to the service life of the restoration. The poor crown margin can easily lead to plaque accumulation and microleakage, which may cause hypersensitivity, caries recurrence, periodontal disease, endodontic lesions, and margin coloring, and then, the service life of the restoration is reduced because of the accelerated decomposition of the adhesive and the reduction in the fracture toughness of the crown due to the increase in its internal gap (Contrepois et al., 2013). Compared with the crown made by the traditional technology, the crown made by 3D printing has a higher degree of fitness to the prepared tooth because it is more accurate (Onoral, 2020). Lee et al. (2017) and Mai et al. (2017) showed that 3D printing was significantly better than CAD/CAM cutting in terms of the suitability of crown margin and interior. Compared with CAD/CAM machining, the 3D printing technology can save materials and produce crowns with high-dimensional accuracy and marginal adaptation within clinically acceptable limits, which has the possibility of clinical application and will become a potential candidate technology for making ceramic restorations (Kim et al., 2017b; Wang and Sun, 2021). For partially worn teeth, veneer restoration is more suitable than complete crown restoration but has higher requirements on the bearing capacity. Ioannidis et al. (2020) have shown that compared with hot-pressed lithium disilicate or CAD/CAM-cutting zirconia occlusal veneers, 3D-printed ultrathin zirconia occlusal veneers exhibit similar or higher bearing capacity and can be used to repair worn teeth.

## 4 Discussion

The biomedical applications of 3D printing are inseparable from the innovation of the 3D printing technology. The accuracy of 3D printing is one of the important factors that make it highly sought after in the field of stomatology, but the accuracy problem will inevitably become the bottleneck for its promotion and development. Accuracy involves two aspects: precision and trueness; precision refers to the closest results under the different replicas by one printing technology, whereas the trueness refers to the closest results of the 3D-printed models and the reference model. Msallem et al. (2020) evaluated the dimensional accuracy of 50 mandibular replicas printed by five common printing technologies and showed that the fused filament fabrication printer has the highest overall precision, whereas the SLS printer has the highest overall trueness.

The “step effect” caused by the layered processing mechanism of 3D printing has an impact on the precision of objects. At present, the mainstream 3D-printing layer thickness is about 0.1 mm, so it is a current research direction to improve the accuracy by reducing the layer thickness to reduce the step. But some studies have shown reducing the layer thickness does not necessarily lead to more precise prints but certainly to longer printing times, which can lead to an increased error rate, raising the probability of print failures. The layer thickness improves the transitions on the diagonals but has little effect on vertical and horizontal edges. Therefore, a precise planning phase with regard to the geometry to be printed is required (Hatz et al., 2020). The DLP device uses a wavelength of 385 nm, and the SLA printing device uses 405 nm; the polymerization efficacy might be impaired because of the different wavelengths, thus increasing dimensional errors within the template. Simultaneously, supports are necessary for the layer-by-layer processing of some 3D printing technologies such as SLA and SLM, whether the additional supports are reasonable or not and the removal of supports will affect the accuracy implicitly. Therefore, the algorithm of additional supports needs to be improved at present.

Despite the existing statistical significance of the differences in accuracy between all the 3D printing technologies studied, when regarded separately, all are minor and competent for clinical applications (Gottsauner et al., 2021). However, the used printing material limits the field of application. When choosing a 3D printer, it is a trend to pay more attention to printing materials in relation to the desired application and the total budget available rather than printing technology. At present, the commonly used 3D printing materials in oral clinics include metals, polymer materials, and ceramics, while more diverse materials are needed in clinical work. The metamorphic sign of metal alloys such as titanium alloys and nickel–cobalt alloys during heat treatment, the biocompatibility of polymer materials, the internal stress, and volume shrinkage of ceramic materials will affect the performance and application of the material, and most of the new materials are still in the *in vitro* test stage without being put into clinical practice due to the physical and chemical properties, and biocompatibility still needs further research. As far as artificial teeth are concerned, there is no material matching all the properties of human teeth at present. In addition, for removable partial dentures, which are made of different materials and artificial teeth, the 3D printing process cannot be performed at one time. If we can find a material that is suitable for 3D printing and matches the teeth more perfectly through the optimization of the composition ratio and processing technology, it will be a great boost to the development of 3D printing.

As one of the high-cost new technologies, the cost issue is usually a concern when introducing it to medical practice. Previously, because of the limitation of expensive 3D printing cost, only professional and private manufacturers were able to produce 3D-printed medical equipment at a reasonable cost under the best conditions. At the same time, due to the late start of 3D printing in China, various materials and instruments are mostly imported, which has a certain restrictive effect on the development of 3D technology in the field of stomatology and even medicine. But it has changed with the advent of cheaper 3D printers and user-friendly 3D software, allowing more and more medical institutions to produce 3D objects. In the opinion of the authors, reducing costs by sharing hardware, software, and materials among medical teams is the best way to promote the 3D printing technology rapidly in the medical field. However, it is necessary to consider stricter regulation of 3D printing in terms of laws and regulations.

## 5 Conclusion

After decades of development, 3D printing technology has become an increasingly important technology in stomatology. At present, 3D printing digital impression technology, guide technology, and bone graft have achieved satisfactory results in various fields of stomatology. With the continuous improvement of precision, the replacement of traditional manufacturing methods by 3D printing will become a reality in the near future.
